# An integrative, peer‐reviewed and open‐source cooperative‐breeding database (Co‐BreeD)

**DOI:** 10.1111/1365-2656.70154

**Published:** 2025-10-23

**Authors:** Yitzchak Ben Mocha, Maike Woith, Sophie Scemama de Gialluly, Lucia Bruscagnin, Natalie Kestel, Shai Markman, Szymon M. Drobniak, Vittorio Baglione, Jordan Boersma, Laurence Cousseau, Rita Covas, Guilherme Henrique Braga de Miranda, Cody J. Dey, Claire Doutrelant, Roman Gula, Robert Heinsohn, Oded Keynan, Sjouke A. Kingma, Ana V. Leitão, Jianqiang Li, Lindelani Makuya, Kyle‐Mark Middleton, Stephen Pruett‐Jones, Andrew N. Radford, Carla Restrepo, Dustin R. Rubenstein, Carsten Schradin, Jörn Theuerkauf, Miyako H. Warrington, Dean A. Williams, Iain A. Woxvold, Michael Griesser

**Affiliations:** ^1^ Department of Biology University of Konstanz Konstanz Germany; ^2^ Zukunftskolleg University of Konstanz Konstanz Germany; ^3^ Centre for the Advanced Study of Collective Behaviour University of Konstanz Konstanz Germany; ^4^ Department of Biology and the Environment University of Haifa Haifa Israel; ^5^ Institute of Environmental Sciences Jagiellonian University Kraków Poland; ^6^ School of Biological, Environmental and Earth Sciences University of New South Wales Sydney Australia; ^7^ Departamento de Biodiversidad y Gestión Ambiental Universidad de León León Spain; ^8^ Cornell Lab of Ornithology Cornell University Ithaca New York USA; ^9^ Centre for Research on Ecology, Cognition and Behaviour of Birds Ghent University Ghent Belgium; ^10^ Laboratório Associado, CIBIO‐InBio, Centro de Investigação em Biodiversidade e Recursos Genéticos University of Porto Vairão Portugal; ^11^ FitzPatrick Institute of African Ornithology, DST‐NRF Centre of Excellence University of Cape Town Rondebosch South Africa; ^12^ Police College National Police Academy Brasília DF Brazil; ^13^ Environment and Climate Change Canada, Wildlife and Landscape Science Directorate Thunder Bay Ontario Canada; ^14^ CEFE, Université Montpellier, CNRS, EPHE, IRD Montpellier France; ^15^ Museum and Institute of Zoology, Polish Academy of Sciences Warsaw Poland; ^16^ Fenner School of Environment and Society The Australian National University Canberra Australian Capital Territory Australia; ^17^ Dead Sea & Arava Science Centre—Masada National Park Hazeva Israel; ^18^ Behavioural Ecology Group, Department of Animal Sciences Wageningen University & Research Wageningen The Netherlands; ^19^ School of Ecology and Nature Conservation Beijing Forestry University Beijing China; ^20^ Université de Strasbourg, CNRS, IPHC UMR 7178 Strasbourg France; ^21^ School of Animal, Plant and Environmental Sciences University of the Witwatersrand Johannesburg South Africa; ^22^ Department of Ecology and Evolution University of Chicago Chicago Illinois USA; ^23^ School of Biological Sciences Bristol UK; ^24^ Department of Biology University of Puerto Rico at Rio Piedras San Juan Puerto Rico; ^25^ Department of Ecology, Evolution and Environmental Biology Columbia University New York USA; ^26^ School of Biological and Medical Sciences Oxford Brookes University Oxford UK; ^27^ Luondu Boreal Research Station Arvidsjaur Sweden; ^28^ Department of Biology Texas Christian University Fort Worth Texas USA; ^29^ Terrestrial Vertebrates Australian Museum Research Institute Sydney New South Wales Australia; ^30^ Department of Collective Behavior Max Planck Institute of Animal Behaviour Konstanz Germany

**Keywords:** alloparental care, birds, comparative research, humans, mammals, open source, repository, social evolution

## Abstract

1. Large‐scale, cross‐species comparative analyses on cooperative breeding—where individuals care for the offspring of other group members—are important for understanding sociality and cooperation. However, the datasets that facilitate these analyses are often limited in precision. To advance comparative research on cooperative breeding, we hereby introduce the Cooperative‐Breeding Database (Co‐BreeD) for birds and mammals.
2. We describe key features of Co‐BreeD's structure: (i) integration of complementary datasets, each presenting a biological parameter relevant to cooperative‐breeding research; (ii) sample‐based (i.e. multiple samples per species linked to an exact sampling location and period); and (iii) open‐source. Respectively, these features enable: (a) comprehensive identification of cooperative‐breeding species according to the user's chosen definition, (b) linking intra‐ and inter‐specific variation in traits with fine‐scale environmental parameters and (c) enabling the research community to correct and expand this database.
3. We present the initial Co‐BreeD dataset, which estimates the prevalence of breeding events involving potential alloparents in 460 populations of 324 species, including 6 human populations (No. total = 43,247 breeding events).
4. We conclude by demonstrating: (i) how Co‐BreeD can improve comparative research (e.g. by enabling the study of cooperative breeding as a continuous rather than a binary trait); and (ii) that cooperative breeding is probably more prevalent than previously estimated in birds and mammals.

## Graphical abstract

Co‐BreeD is the first updatable and open‐source database dedicated to cooperative breeding. It enables continuous, non‐binary measurement of cooperative breeding, transforming how we study breeding systems across taxa. Further, by linking biological estimates to their exact sampling location and period, it allows for unprecedented accuracy in the comparative study of the ecology of sociality.

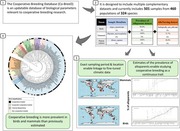

## Abstrakt

1. Bei der kooperativen Jungenaufzucht beteiligen sich Gruppenmitglieder an der Versorgung von Nachwuchs, der nicht ihr eigener ist. In bisherigen Datenbanken erfolgte die Klassifikation meist binär: Kooperativ—ja oder nein. Diese Vereinfachung wird der biologischen Vielfalt jedoch nicht gerecht, denn bereits innerhalb einer Art kann es zu erheblichen Unterschieden kommen. Das erschwert sowohl innerartliche als auch übergreifende Vergleiche zwischen Arten und Taxa.
2. Mit Co‐BreeD (Cooperative‐Breeding Database) stellen wir eine neue Datenbank vor, die populationsspezifische Informationen zur kooperativen Jungenaufzucht bei Vögeln und Säugetieren dokumentiert. Im Gegensatz zu bisherigen Systemen erlaubt sie eine differenzierte Erfassung innerhalb einer Art und ist so angelegt, dass zukünftige Ergänzungen und Korrekturen problemlos integriert werden können.
3. Hier wird der erste Datensatz von Co‐BreeD vorgestellt. Er erfasst, bei wie vielen Reproduktionsereignissen—etwa Gelege, Bruten oder Würfen—Gruppenmitglieder aktiv an der Jungenaufzucht beteiligt waren. Insgesamt enthält der Datensatz Angaben zu 460 Populationen aus 324 Arten und umfasst 43.247 Reproduktionsereignisse.
4. Unsere Ergebnisse zeigen: (i) Die Struktur von Co‐BreeD ermöglicht erstmals die Analyse kooperativer Jungenaufzucht als kontinuierliche Variable statt als binäres Merkmal. Dadurch verbessert sich die Qualität von Vergleichsstudien erheblich. (ii) Kooperative Jungenaufzucht tritt bei Vögeln und Säugetieren häufiger auf als bislang angenommen—auch bei Arten, die zuvor nicht als kooperativ klassifiziert wurden, zeigen sich Hinweise auf Beteiligung von Gruppenmitgliedern an der Aufzucht.

## 1 INTRODUCTION

Cooperative breeding is an offspring care system (Kappeler, 2019) in which individuals provide alloparental care to the young of other group members (Ben Mocha, Scemama de Gialluly, et al., 2023; Brown, 1974; Stacey & Koenig, 1990). Ever since Darwin (1888), this phenomenon has attracted continuous and interdisciplinary interest (Boland & Cockburn, 2002; Nyaguthii et al., 2025; Skutch, 1935). For instance, behavioural ecologists have examined why individuals invest in others' fitness (Macleod et al., 2015; Sherman et al., 1995; Zahavi & Zahavi, 1997), comparative psychologists have investigated whether alloparental care facilitates complex social cognition (Burkart & van Schaik, 2010; Tomasello & Gonzales‐Cabrera, 2017), and as humans are cooperative breeders, anthropologists have endeavoured to understand this caring system to shed light on our species' evolution (Hill & Hurtado, 2009; Hrdy, 2007).

Large‐scale cross‐species comparisons are powerful tools to investigate the causes and consequences of cooperative breeding (Barsbai et al., 2021; Camerlenghi et al., 2022; Dey et al., 2017; Downing et al., 2020; Kingma, 2017; MacLeod & Lukas, 2014). These analyses use advanced statistical methods that control for phylogenetic relationships when comparing cooperatively versus non‐cooperatively breeding species in relation to potential causes (e.g. climatic variables (Griesser et al., 2017; Jetz & Rubenstein, 2011)) or the consequences of alloparental care (e.g. the prevalence of extra‐group offspring (Cornwallis et al., 2010; Kingma et al., 2010)). The credibility of these analyses, however, depends on the accuracy of the care system (e.g. cooperative vs. non‐cooperative breeder) and the biological and ecological estimates ascribed to each species (e.g. litter size, annual rainfall in habitat). Although existing datasets on bird and mammal care systems have paved the way to an advanced understanding of cooperative breeding, accumulating literature demonstrates certain limitations (reviewed by Ben Mocha, Dahan, et al., 2023; Brouwer & Griffith, 2019; Cockburn, 2020; Griesser & Suzuki, 2016; Schradin, 2017). Cooperative‐breeding datasets often include three major sources of inaccuracy. First, some studies have defined cooperative breeding using vague parameters (e.g. using non‐distinct quantitative terms such as ‘regularly’, or not defining what constitutes ‘alloparental care’) (reviewed by Ben Mocha, Scemama de Gialluly, et al., 2023; Lukas & Clutton‐Brock, 2017). Second, comparable assessment of socio‐biological parameters across bird and mammal species often requires taxon‐based adjustments (e.g. what should be considered a breeding event in each species? see Section 3.1.3). Yet, biologically meaningful adjustments are unlikely to be accomplished without assistance from species‐specific experts (see Section 2.10). Third, incorrect information, such as typographic errors in numeric values (reviewed by Brouwer & Griffith, 2019), is often perpetuated since paper‐associated datasets are rarely checked for accuracy, corrected or updated.

In addition, most current datasets are somewhat limited in scope. First, species are usually represented by a single population, thereby hindering exploration of within‐species variation and being prone to random sampling errors of a potentially unusual population (Barsbai et al., 2021; Brouwer & Griffith, 2019). Second, most comparative datasets focus on one taxon (i.e. birds, non‐human mammals or humans), thus preventing a more comprehensive study of cooperative breeding. Third, datasets rarely include comparative data from human populations, thereby hampering research about human evolution within the broader cooperative‐breeding framework (but see Ben Mocha, 2020; Burkart et al., 2017). As a result, a growing number of scholars are calling for more refined and transparent data curation (Ben Mocha, Scemama de Gialluly, et al., 2023; Brouwer & Griffith, 2019; Griesser & Suzuki, 2016; Huck et al., 2020; Lukas & Clutton‐Brock, 2017; Schradin, 2017).

Here, we introduce the Cooperative Breeding Database (Co‐BreeD), a multi‐dataset open‐source resource designed to advance the comparative study of cooperative breeding. In Section 2, we provide a methodological account of Co‐BreeD's structure and explain how it is designed to overcome the above‐mentioned limitations. In Section 3, we present a methodological account of the first dataset curated to Co‐BreeD: the Prevalence of breeding events with potential Alloparents (*PA*). We conclude with a descriptive summary of the *PA* dataset and a discussion about how Co‐BreeD can facilitate a more comprehensive identification of cooperative‐breeding species and the study of cooperative breeding as a continuous trait.

## 2 Co‐BreeD: COOPERATIVE‐BREEDING DATABASE

Co‐BreeD is a growing database curating population‐level estimates of biological parameters relevant to the research of cooperative‐breeding birds and mammals (e.g. the prevalence of alloparents, allonursing). Below, we discuss its key features.

### 2.1 FAIR: Findable, Accessible, Interoperable, Reusable

Co‐BreeD fulfills the FAIR principles for scientific data management (Wilkinson et al., 2016). Findable: a folder with all the Co‐BreeD project files is stored in the Zenodo repository (including the database metadata: https://doi.org/10.5281/zenodo.14697198 (Ben Mocha, 2025)).

Accessible: The Co‐BreeD folder includes three files: (i) the database file in RData format (R Core Team, 2022); (ii) R code for quality check (see below); and (iii) a data submission form for data contributors. All Co‐BreeD files are of a non‐proprietary format and are stored in an open‐access repository. The Co‐BreeD RData file contains the entire database without users needing to submit usage requests.

Interoperable: Co‐BreeD is designed to integrate multiple datasets (each dedicated to a different biological parameter), and each future dataset will be accompanied by a methodological account (e.g. Section 3 of this paper). The index/data submission form has a How‐To section with a detailed explanation of each data column and instructions for data contributors.

**FIGURE 1:**
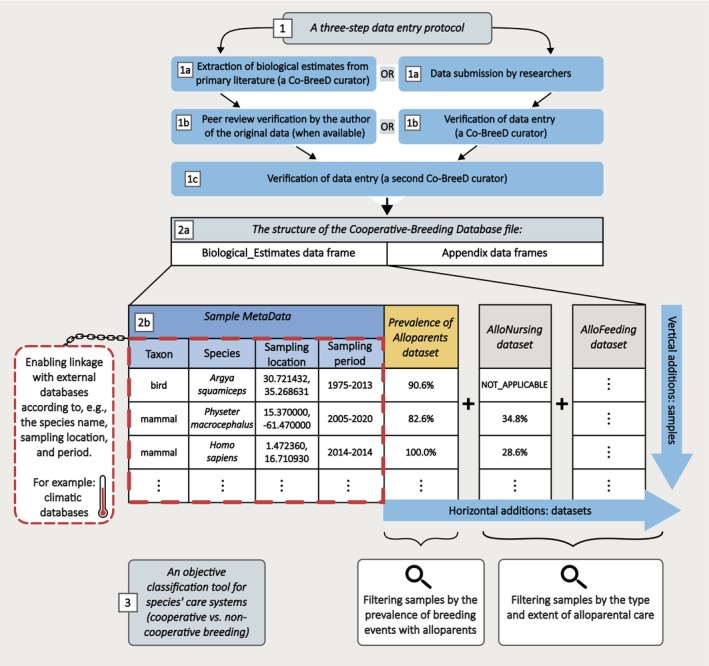
The Cooperative‐Breeding Database (Co‐BreeD). (1) Data entry protocol. (2a) The structure of the Co‐BreeD RData file. (2b) The horizontal and vertical structures of Biological_Estimates data frame. (3) Co‐BreeD enables filtering species/samples according to the conditions set by different cooperative‐breeding definitions. The Prevalence of Alloparents (*PA*) dataset is published in this article; other datasets are under preparation (e.g. *AlloNursing*).

Reusable: The Sample MetaData section in Co‐BreeD provides the metadata for each sample. It ensures the replicability of Co‐BreeD's biological estimates by providing the title, DOI, and the page/table/figure number of the scientific source from which the biological estimates were extracted. Further, the metadata specifies the exact coordinates of the sampling location (WGS 84 format) and the sampling period of each sample. This information enables researchers to link Co‐BreeD's biological estimates with other databases that include information from the same location and period (e.g. climatic data, species distribution, Figure 1).

### 2.2 Database structure

The database file includes two types of R data frames (Figure 1, section 2a). The main data frame is named ‘Biological_Estimates’. It includes the Sample MetaData section and multiple datasets (see next paragraph, Figure 1, section 2b). The second type of data frame is the appendices that include a breakdown of information of some datasets in the Biological_Estimates data frame (e.g. the current PA_appendix, see Section 3).

### 2.3 Horizontal structure: Integration of multiple datasets

The Biological_Estimates data frame has a multi‐dataset horizontal structure (columns) and a sample‐based vertical structure (rows, Figure 1). Its multi‐dataset structure begins with the Sample MetaData section, where a cluster of 16 columns provides the metadata of the sample (e.g. the sampling location and period). This section is followed by a horizontal sequence of datasets, each consisting of a cluster of columns providing information on a specific biological parameter. The hereby published initial Co‐BreeD dataset presents estimates of the prevalence of breeding events with potential alloparents in a population (Section 3). Additional datasets that are currently being curated are estimates of allonursing and litter/brood size. To facilitate data processing, each column header includes a suffix according to the dataset it belongs to. For example, the columns of the Sample MetaData section have the suffix ‘SMD’ and the columns of the Prevalence of breeding events with potential Alloparents dataset have the suffix ‘PA’.

### 2.4 Vertical structure: Sample‐based

The Biological_Estimates data frame has a sample‐based structure, where each row represents a sample of breeding events of a specific species from a defined sampling location and period. The multi‐dataset structure of Co‐BreeD enables new data sets (i.e. additional biological parameters) to be connected with the other biological parameters extracted from the sample via the Sample MetaData. Having a sample‐based structure also allows the inclusion of samples from multiple populations of a species and/or multiple sampling periods for the same population. Linkage between the sample‐based biological estimates in Co‐BreeD and fine‐scale environmental data (that can be extracted from external databases according to the sampling location and period) enables accurate investigation of (i) spatial variability (e.g. between populations (Balmforth, 2004; Brouwer & Griffith, 2019)) and (ii) temporal variability (within population (Curry & Grant, 1989; Heinsohn et al., 2000)) with unprecedented accuracy.

### 2.5 ‘Open‐source’ and updatability

Co‐BreeD favours a relatively small number of accurate data estimates over a larger sample size with potentially less reliable estimates (see Figure 5 for Co‐BreeD's criteria for data inclusion/exclusion). This preference is advocated by behavioural ecologists (Griesser & Suzuki, 2016; Heldstab et al., 2017; Schradin, 2017) and statisticians (Bradley et al., 2021; Meng, 2018) who demonstrated that smaller samples of accurate data are more informative than larger samples of less accurate data. Since the care system of most bird and mammal species is currently unknown or only indirectly inferred (Cockburn, 2006; Griesser & Suzuki, 2016; Lukas & Clutton‐Brock, 2017; Schradin, 2017), curating a reliable database of the care systems of all bird and mammal species is impossible at present.

To address this limitation, Co‐BreeD is designed as an ‘open‐source’ resource to which corrections and additions can be made. This can be done in two ways. First, by hereby publishing the Co‐BreeD database alongside a full methodological account and R code that ensures format consistency (see below), we enable every person to build on our database and expand it with new samples and/or datasets on new biological parameters (as a new file rather than an official updated version of Co‐BreeD). Second, the corresponding author of this paper plans to publish new Co‐BreeD datasets alongside a methodological paper describing each of them. These datasets will be published in a new official Co‐BreeD R file (under a version‐unique DOI) containing all previously published datasets in addition to the new dataset, including corrections to previous datasets.

### 2.6 Objective classification tools

**FIGURE 2:**
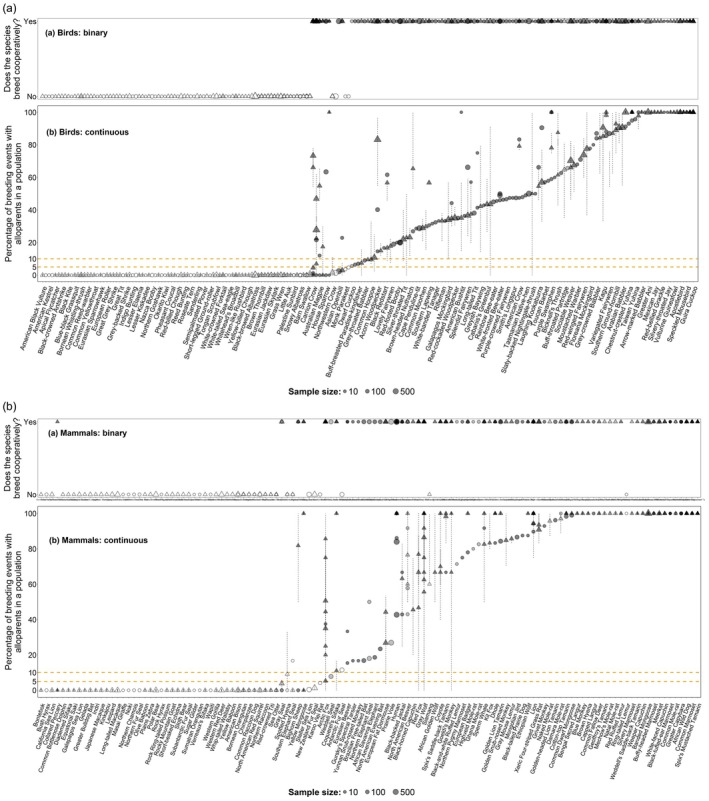
(a) Binary species classification as cooperative vs. non‐cooperative breeders; and (b) Continuous estimates of the proportion of breeding events with potential alloparents of the same bird and mammal species included in the Co‐BreeD *Prevalence of Alloparents* (*PA*) dataset. No. birds = 286 samples, 266 populations, 203 species; No. mammals = 215 samples, 194 populations, 121 species. For clarity, every other name is omitted from the *x*‐axis on the bird figure (see Figure S1 for all species names). Species are presented in ascending order, starting with the species with the lowest prevalence of breeding events with potential alloparents. Circles represent the percentage of breeding events with potential alloparents in a specific sample. A triangle within a line represents the percentage (triangle) and multi‐year range (line) for the same population. The symbols' size reflects sample size (limited to 500 breeding events). Darker shapes indicate overlapping estimations from multiple samples. Empty shapes represent samples from non‐cooperatively breeding species (i.e. where, to the best of our knowledge, no population exhibits a systematic and unequivocal form of alloparental care, such as allonursing, allofeeding or incubation). For clarity, in samples with a multi‐year range of 0%—0% or 100%—100%, the range was extended by one percentage point lower and higher (e.g. −1%—1% instead of 0%—0% in leopard). These manipulated ranges are represented by solid lines. The orange dashed lines represent the minimum thresholds suggested to define cooperatively breeding species under various binary classifications (percentage of breeding events with alloparents in at least one population of the species: >5% (Ben Mocha, Scemama de Gialluly, et al., 2023); >10% (Cockburn, 2006)).

**FIGURE 3:**
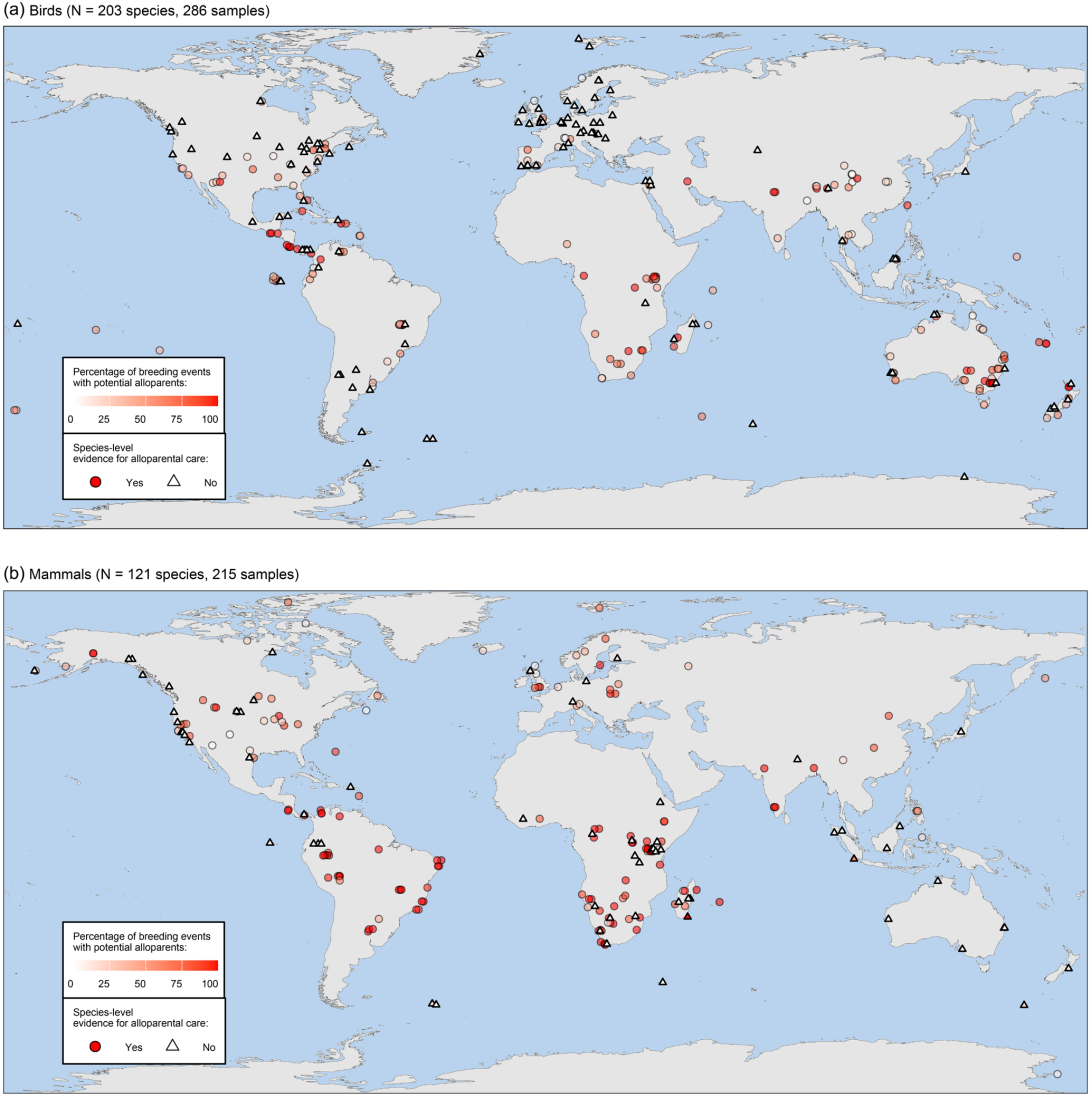
The geographical distribution of (a) bird and (b) mammal samples included in the Co‐BreeD *Prevalence of Alloparents* (*PA*) dataset. Each shape represents a sample from a specific sampling location and period. Locations were jittered to present overlapping samples.

Co‐BreeD offers two unique classification capabilities. First, by providing continuous estimates of biological parameters, it enables the study of cooperative breeding as a continuous trait (e.g. using the percentage of breeding events with alloparents in the population as the response variable). For instance, in a binary classification system, the black catbird *Melanoptila glabrirostris* is a non‐cooperatively breeding species, and the long‐tailed tit *Aegithalos caudatus* and greater ani *Crotophaga major* are both cooperatively breeding species. However, the ‘degree’ of cooperative breeding may also be viewed as a continuous trait, described by the proportion of breeding events in a population that are attended by alloparents (black catbird population in Mexico—0% (Lapergola, 2012), long‐tailed tit population in the UK—54% (Hatchwell & Russell, 1996), greater ani population in Panama—100% (Riehl & Smart, 2022)). Studying cooperative breeding as a continuous trait accounts for the substantial variation observed in the occurrence of alloparental care between and within species (Figures 2 and 3) and provides stronger explanatory power than binary classification (Clutton‐Brock, 2021; Olivier et al., 2024).

Second, Co‐BreeD facilitates a more accurate binary classification of species as cooperatively versus non‐cooperatively breeders. By curating complementary datasets of biological traits previously proposed to define cooperative breeding, Co‐BreeD enables users to filter species based on the criteria required by their chosen definition. For example, Co‐BreeD allows filtering for species with >5% (Ben Mocha, Scemama de Gialluly, et al., 2023) or >10% (Cockburn, 2006; Cornwallis et al., 2010) of breeding events involving alloparents. Yet, the Biological_Estimates data frame also suggests a species‐level classification according to Ben Mocha, Scemama de Gialluly, et al. (2023) binary definition of cooperative breeding.

### 2.7 Statistical accounting for sampling error probability

**FIGURE 4:**
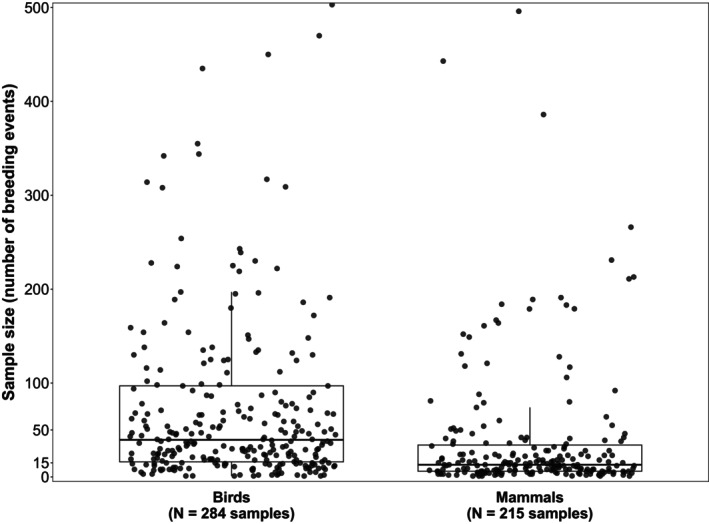
Sample sizes on which the estimations of the Co‐BreeD *Prevalence of Alloparents (PA)* dataset are based. Horizontal bars represent the medians, boxes the 25% and 75% quartiles and whiskers extend to the minimum and maximum values within 1.5 times the interquartile range. Data points outside the whiskers are outliers. Each jittered circle represents a sample. For clarity, 16 maximal outlier samples that were used to calculate the boxplots were excluded from the bird plot (range: 503–2792 breeding events).

Data on parental care is often based on small sample sizes (Cockburn, 2006; Schradin, 2017), making them prone to higher sampling error. Co‐BreeD addresses this issue by providing the sample size for each estimate (Figure 4) and, for some samples, a year‐by‐year breakdown of data. This set of values enables weighted analyses that account for variation in the statistical power between samples.

### 2.8 Included species

**FIGURE 5:**
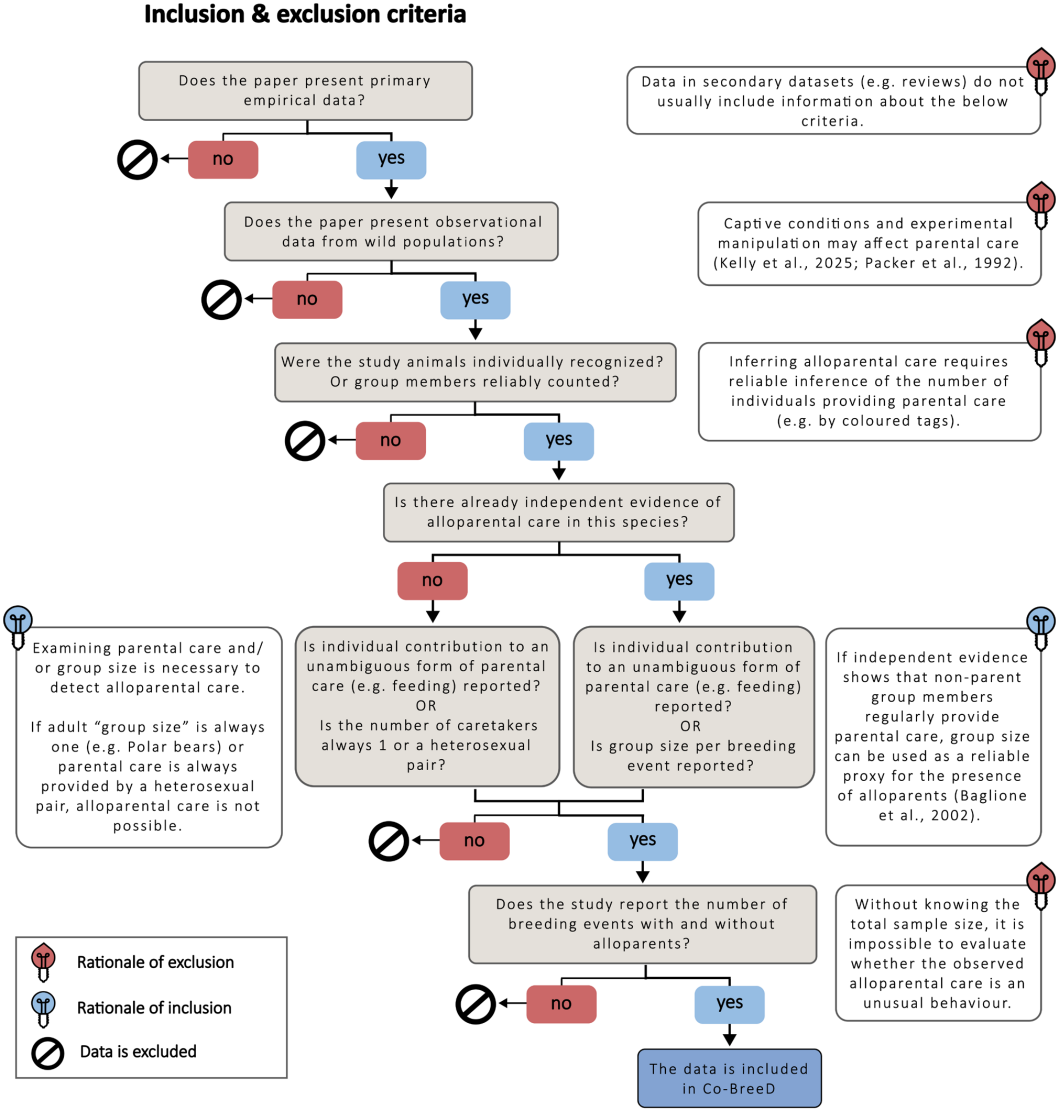
A flowchart of the inclusion and exclusion criteria of data used in the Prevalence of Alloparents (*PA*) dataset.

Resource‐dependent, Co‐BreeD aims ultimately to include as many bird and mammal species with qualified data (Figure 5). Species are included regardless of whether they are cooperative breeders or not (i.e. species that do not exhibit systematic and unequivocal forms of alloparental care such as incubation, nursing, or feeding by group members other than the parents). The inclusion of non‐cooperatively breeding species enables contrast with cooperative breeders, which is critical to identify the causes and consequences of cooperative breeding. Co‐BreeD focuses on birds and mammals due to limited resources and the conceptual complexity of developing a valid comparative framework across numerous taxa. Species nomenclature follows the Handbook of the Birds of the World (HBW and BirdLife International, 2024) and the Mammal Diversity Database (Mammal Diversity Database, 2024).

### 2.9 Natural variation

Co‐BreeD estimates natural variation in alloparental care. It thus only includes data from free‐living populations and excludes data from experimental studies where manipulation might affect parental care (Kelly et al., 2025; Packer et al., 1992).

### 2.10 Reviewing and archiving unpublished data

While Co‐BreeD focuses on extracting biological estimates from peer‐reviewed published literature, it accepts submissions of unpublished data if (i) the methods used were published elsewhere, (ii) Co‐BreeD curators reviewed the unpublished data and (iii) the data are allowed to be published in Co‐BreeD. By archiving unpublished behavioural datasets from populations whose habitats have been substantially modified after data collection, Co‐BreeD preserves knowledge about behaviours that may be adaptive in these pre‐modified habitats (Kühl et al., 2019; Neyman, 1978).

### 2.11 Quality control and mutual peer review

To minimise the risk of transcription errors (e.g. typos) and subjective judgement, Co‐BreeD has a three‐step data entry protocol (Figure 1). First, biological estimates are calculated from the primary literature by a Co‐BreeD curator. Second, the data entry is reviewed by another Co‐BreeD curator (including replicating the calculation of estimates from the cited resource). Third, if available, the author of the publication from which the estimates were calculated is asked to peer review our data entry and to provide missing data (e.g. a year‐by‐year breakdown of the data). Alternatively, data are initially contributed by scholars and are then peer‐reviewed by two Co‐BreeD curators. Co‐BreeD thus implements a mutual peer‐review process, in which curators review published data or data submitted by field researchers, while the author reviews the corresponding Co‐BreeD data entry.

To increase transparency and automatic reproducibility (Wilkinson et al., 2016), the Co‐BreeD folder includes a quality control code to check common transcription errors in each column.

### 2.12 Adjustment to species‐specific characteristics

In‐depth familiarity with a species is important as species‐specific traits may require a nuanced approach to calculating socio‐biological estimates (Brouwer & Griffith, 2019; Lukas & Clutton‐Brock, 2017). For example, the basic social unit from which reproductive skew indices should be calculated differs according to whether a species lives in discrete groups or in multi‐level societies (Ben Mocha, Dahan, et al., 2023; Painter et al., 2000). Authors of the original publications are thus asked to review the rationales used by Co‐BreeD curators to ensure that the most biologically meaningful estimates are made for each species.

### 2.13 Data submission and fair collaboration

Scholars with data that fulfill Co‐BreeD's inclusion criteria (Figure 5) are welcome to send their data to the corresponding author using the submission form in the Co‐BreeD folder (https://doi.org/10.5281/zenodo.14697198). Co‐BreeD acknowledges the importance of authors verifying their data and contributing further unpublished data. Verifying authors are indicated next to their sample and are invited to co‐author one of Co‐BreeD's methodological accounts.

## 3 THE FIRST Co‐BreeD DATASET: PREVALENCE OF BREEDING EVENTS WITH POTENTIAL ALLOPARENTS ( *PA* )

In this section, we present a methodological account of the first dataset curated to Co‐BreeD: the Prevalence of breeding events with potential Alloparents (*PA*). In species that do exhibit alloparental care, this behaviour often does not occur in all breeding events (i.e. clutches and litters) within a population or among all populations of the species (Figure 2). This variation introduces two problems for comparative research that relies on a binary classification of cooperative vs. non‐cooperative breeders. First, by ignoring this substantial inter‐ and intra‐species variation (Figure 2), binary classification systems diminish explanatory power (Clutton‐Brock, 2021; Olivier et al., 2024). Second, in well‐studied species, rare behaviours (e.g. Nichols & Arbuckle, 2022; Price et al., 1983) or potential misinterpretations (e.g. Carr, 1993; Włodarczyk & Kołaciński, 2001) are likely to be reported. Thus, if a single case of alloparental care is sufficient to classify a species as a cooperative breeder, species will be systematically misclassified as cooperative breeders as research effort increases (false positives). Binary classification is, therefore, a ‘one‐way process’, where species previously labelled as non‐cooperative breeders can be reclassified as cooperative breeders, but are then rarely corrected if accumulating evidence suggests otherwise (Ben Mocha, Scemama de Gialluly, et al., 2023; Griesser & Suzuki, 2016); for instance, when additional observations only record breeding without alloparents.

To address these issues, the first Co‐BreeD dataset provides quantitative estimates of the prevalence of breeding events with potential alloparents. This continuous measure of cooperative breeding (i) enables greater explanatory power and (ii) minimises the impact of rare or mistaken observations of alloparental care by weighting them against the overall prevalence of non‐cooperative breeding events within the population.

The Co‐BreeD *Prevalence of potential Alloparents* dataset consists of 13 columns. The title of each column ends with the suffix ‘PA’ and is explained in the index data frame and data submission template.

### 3.1 Methods

#### 3.1.1 Data collection

Data collection began in 2023 and is ongoing. Relevant natural history data are gathered in two complementary ways that follow the PRISMA‐P protocol (Moher et al., 2015). First, we systematically review the primary literature cited to support the classification of the species included in datasets on bird and mammal care systems (so far reviewed: Ben Mocha, Scemama de Gialluly, et al., 2023; Federico et al., 2020; Lukas & Clutton‐Brock, 2012) and parental care (so far reviewed: Arslan, 2024; Lukas & Clutton‐Brock, 2013; MacLeod & Lukas, 2014; Mitani & Watts, 1997; Packer et al., 1992; Riedman, 1982; Vodenková, 2018). Second, to facilitate a comprehensive representation of taxonomic groups and geographical areas that are under‐represented in the current datasets, we (i) invite experts of these species to submit their data and (ii) search Web of Science, Google Scholar and ResearchGate for the taxon name with one of the following keywords at a time: (geographical location), ‘parental care’, ‘alloparental care’, ‘allonursing’, ‘feeding’, ‘cooperative breeding’ or ‘communal breeding’. To decrease the bias towards studies written in English (Amano et al., 2023), Co‐BreeD curators repeat these targeted searches and review relevant literature in their native languages (mostly French, German and Hebrew). See Figure 5 for the inclusion criteria for studies in the *PA* dataset.

#### 3.1.2 Breeding events with potential alloparents

To estimate the prevalence of breeding events with potential alloparents in a sample, we calculate the percentage of breeding events with potential alloparents out of the total number of breeding events observed. Below, we define ‘breeding event’ and ‘potential alloparent’, and describe how non‐cooperative‐breeding species were assessed.

#### 3.1.3 Breeding events

The brood/litter level is the most basic level of breeding events. It is especially important to consider this level in species where social groups often include multiple breeding females and in species where only some broods/litters within the social group receive alloparental care (Baldovino & Di Bitetti, 2008; Pike et al., 2019). Hence, data permitting, we consider each brood/litter as a distinct breeding event. This means that in social groups with multiple breeding females, we count each brood/litter produced by all the females in the group.

**FIGURE 6:**
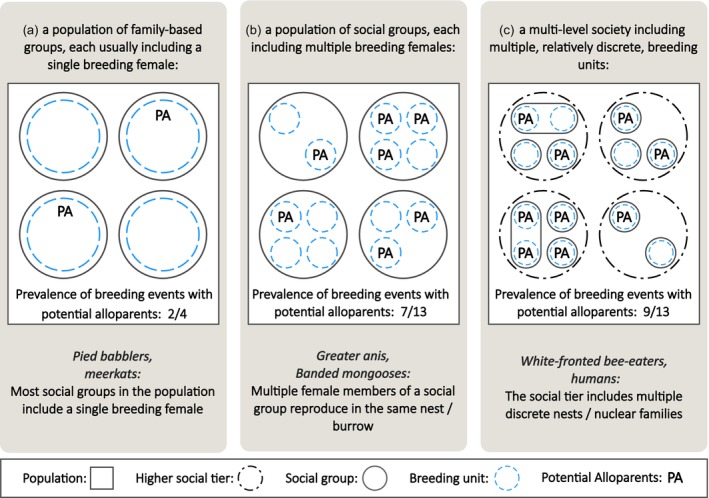
Examples of common social systems in birds and mammals. Each example of a social system presents species in which this social system is most common, although different types of social systems often co‐occur within a population. The dashed blue circles indicate the breeding units used to calculate the proportion of breeding events with potential alloparents.

To clarify what we consider a breeding event, we present examples from three common social systems. First, in family‐based groups where there is often a single breeding female (e.g. southern pied babblers *Turdoides bicolor*; Figure 6a), we consider each brood as one breeding event. Second, in species living in groups with multiple breeding females, we consider the brood/litter of each mother as a breeding event (Figure 6b). For instance, in the multi‐male–multi‐female groups of white‐faced capuchins *Cebus capucinus* (Sargeant et al., 2015), because each capuchin mother typically has only one offspring per birth, we calculate the number of infants receiving alloparental care out of the total number of infants observed in the group. Third, in species living in multi‐level societies, relatively discrete ‘breeding units’ (e.g. pairs, polyandrous families) are clustered and nested together within a social tier that creates a layer in a more complex, stratified social organisation (Figure 6c; Grueter et al., 2012, 2020). In these species, as in the second example, we consider each brood/litter from each breeding unit as a breeding event. Examples include white‐fronted bee‐eater *Merops bullockoides*, where the greater social group (‘colony’) has multiple nests simultaneously (Emlen & Wrege, 1988) and small‐scale human communities consisting of discrete families (Grueter et al., 2012). In both cases, we calculated the percentage of nests/families receiving alloparental care rather than the percentage of social colonies/villages with alloparents, respectively.

The column ‘definition breeding event with potential alloparents_PA’ in the *PA* dataset specifies what was considered a breeding event with potential alloparents for each sample. The ‘type of sample_PA’ column specifies whether the sample included ‘unique breeding units’ (i.e. each breeding event was made by a unique social group or mother) or ‘repeated measurements’ (i.e. at least some social groups or mothers produced multiple breeding events in the sample).

#### 3.1.4 Potential alloparents

Following the distinction between parental care systems and mating systems (Kappeler, 2019; Kappeler & van Schaik, 2002), we consider individuals as alloparents if they provide care to other group members' offspring, regardless of whether the alloparent breeds at the same time or not (Ben Mocha, Dahan, et al., 2023; Cockburn, 2006). Consequently, we use the term alloparents instead of helpers to avoid implying that individuals who take care of others' offspring are necessarily non‐breeders. When data on the number of broods/litters was unavailable, the number of breeding females per group‐year was used as a proxy for breeding events (because all broods/litters are produced by females, including those with shared paternity).

To exclude cases where care is provided for extra‐group offspring⸺as in brood parasitism, extra‐pair and extra‐group paternity⸺we mostly considered cases where alloparents care for the offspring of their social group members (see discussions in Ben Mocha, Scemama de Gialluly, et al., 2023; Griesser & Suzuki, 2016). An exception was made for colonial breeders whose young sometimes receive care by other colony members besides their parents (hereafter ‘colonial alloparenting’. e.g. pinnipeds (Riedman & Le Boeuf, 1982) and king penguins *Aptenodytes patagonicus* (Lecomte et al., 2006)).

We present data on breeding events with ‘potential’ alloparents to acknowledge that alloparental care is sometimes reliably assumed according to specific criteria rather than through direct observation of alloparenting. For example, for species where alloparenting is virtually always provided by all adult group members, some studies have assumed that active alloparents are present in all breeding groups with more adults than the heterosexual pair (Baglione et al., 2002; Roldán et al., 2013). As a rule, group size was considered a proxy for breeding events with or without potential alloparents: (i) for species with strong previous evidence for systematic alloparental care; and (ii) for samples where alloparental care can be reliably ruled out, such as where all observed breeding events had a single caretaker (e.g. polar bears *Ursus maritimus* (Ramsay & Stirling, 1986)), or where alloparenting is unlikely, such as where breeding groups comprise a heterosexual pair of caretakers only (Monk parakeet *Myiopsitta monachus* (Bucher et al., 2016)). Estimates that are based on a proxy of alloparental care are specified in the ‘violated criteria_PA’ column (code: parental_care) and should be treated with caution (this column also indicates samples where not all animals could be recognised individually (code: individual_recognition)).

#### 3.1.5 Assessment of non‐cooperatively breeding species

Identifying non‐cooperative‐breeding species requires confirming the absence of alloparental care. To this end, we review studies on at least one form of care (e.g. nursing, feeding) provided by parents in the focal species and define breeding events with potential alloparents as those where this form of care was provided by individuals other than the parents. We assume that if such cases of alloparenting were not reported by the authors, then they were not observed. For instance, for a study examining the feeding of nestlings by individually marked Palestine sunbirds *Cinnyris osea* (Markman et al., 2002), a case of >2 birds feeding the nestlings would have been considered a brood with alloparents. However, since the authors did not report such a case, we assumed it was not observed.

### 3.2 Results and discussion

#### 3.2.1 Sample

**FIGURE 7:**
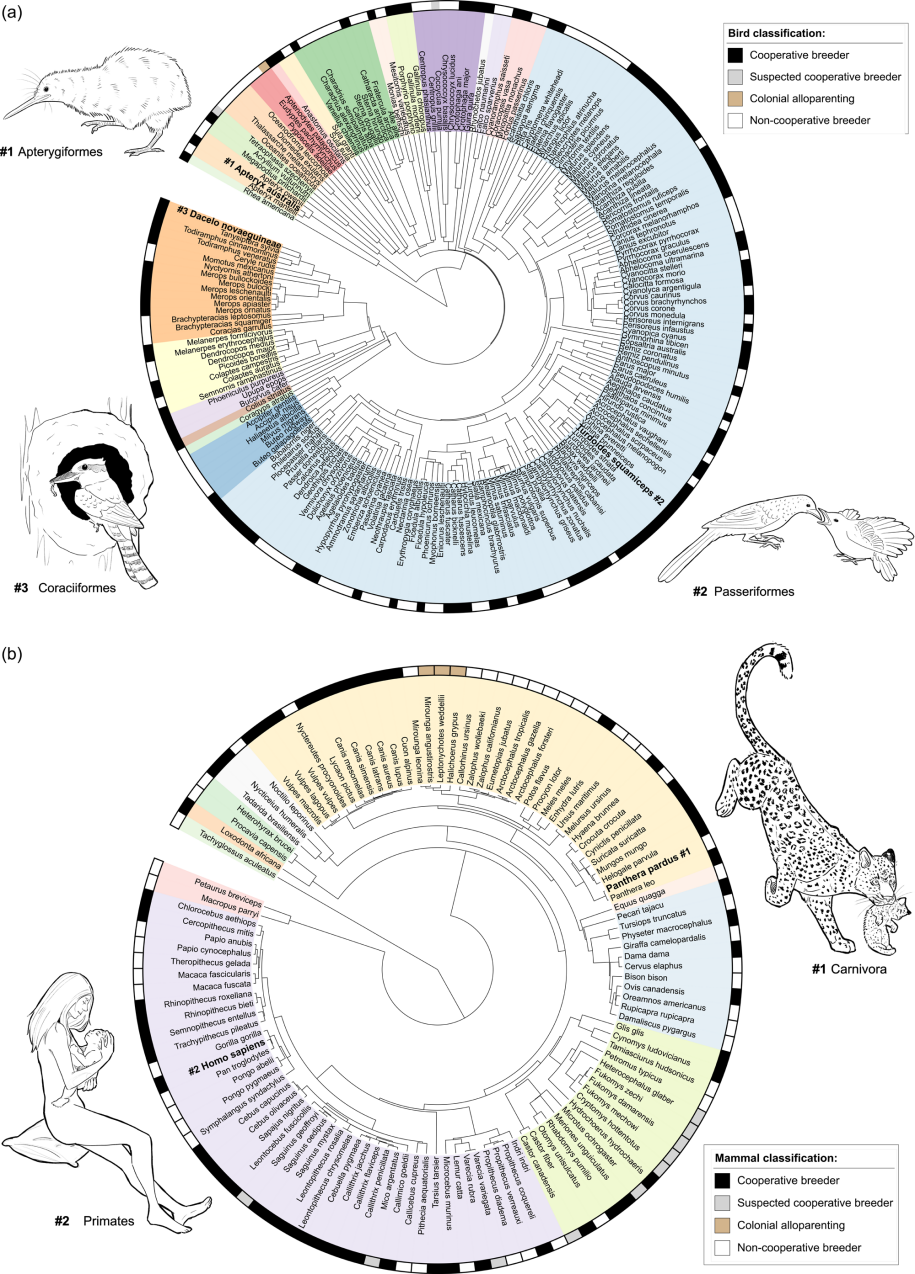
Phylogenetic trees of the (a) bird and (b) mammal species in Co‐BreeD. Taxonomic orders are marked by different colours. Phylogenetic trees were made in phyloT and visualised using iTOL (Letunic & Bork, 2024). Animal illustrations: Natalie Kestel.

The *PA* dataset currently documents over 43,247 breeding events from 501 samples (Table 1). Taxonomically, these samples are distributed across 21 out of the 37 bird orders (HBW and BirdLife International, 2024) and 10 of the 27 mammal orders (Mammal Diversity Database, 2024; Figure 7). Geographically, Co‐BreeD samples are distributed across all seven continents (Figure 3).

**TABLE 1:** Sample size of the Co‐BreeD *Prevalence of Alloparents* dataset (as of September 2025).

	Number of species	Number of populations	Number of samples	Samples confirmed by the original authors
Birds	203	266	286	22%
Non‐human mammals	120	188	208	24%
Humans	1	6	7	57%
Total	324	460	501	23% (+15% of samples that could not be verified)

#### 3.2.2 Directing research effort

Estimates of parental care are often based on small sample sizes (median breeding events in mammals: 13 [range: 1–2201] and birds: 40 [range: 1–2792]; Figure 4). Additional research that would increase sample size is needed to ensure robust conclusions, especially in mammals.

#### 3.2.3 Intraspecific variation

The Co‐BreeD *PA* dataset facilitates research on intraspecific variation across time and space by providing: (i) a multi‐year range for 299 samples from 217 species (Figure 2), (ii) estimates from multiple populations of 83 species (range: 2–9 populations per species; Figure 2) and (iii) 193 year‐by‐year datasets from 142 species (PA_appendix).

Of the 83 species with estimates from more than one population, 13% include populations both under and above the 5% threshold of breeding events with alloparents used by some binary cooperative‐breeding definitions (Ben Mocha, Scemama de Gialluly, et al., 2023; Cockburn, 2006); for example, ground tit *Pseudopodoces humilis*, greater rhea *Rhea americana* and yellow mongoose *Cynictis penicillata* (Figure 2). In these species, the binary classification as cooperative or non‐cooperative breeders would depend on the population sampled. This intraspecific variation exemplifies the importance of studying cooperative breeding at the population level and as a continuous trait; thereby removing the need for minimum thresholds in cooperative‐breeding definitions.

#### 3.2.4 Cooperative breeding is more prevalent than previously recorded

We classified 55% of the 303 species included in Co‐BreeD as cooperative breeders (excluding 21 ambiguous species). That is, at least one wild population of these 55% species exhibits systematic and unequivocal forms of alloparental care such as incubation, nursing, feeding or transporting of young by group members other than the parents (Ben Mocha, Scemama de Gialluly, et al., 2023).

Currently, cooperatively breeding species are overrepresented in Co‐BreeD since we have so far reviewed datasets that focus on alloparental care. However, Co‐BreeD can already be used to examine the accuracy of current estimates for cooperative breeding in birds (3.6% (Downing et al., 2020); 9% (Cockburn, 2006); 14% (Griesser et al., 2017)) and mammals (i.e. cooperative and so‐called communal breeding mammals: 3.1% (Lukas & Clutton‐Brock, 2012); only in carnivores: 12.3% (Federico et al., 2020)). Table 2 demonstrates that most species previously classified as cooperative breeders are also classified as cooperative breeders in Co‐BreeD. By contrast, Co‐BreeD reports evidence for alloparental care in 13%–17% of the bird species and 9–30% of the mammal species previously classified as neither cooperative nor communal breeders (Table 2).

**TABLE 2:** The number of species classified in Co‐BreeD as having species‐level evidence for cooperative breeding (i.e. species exhibiting systematic alloparental care in >5% of breeding events in ≥1 population) and species classified as cooperative/communal breeders in bird (Cockburn, 2006; Downing et al., 2020) and mammal (Federico et al., 2020; Lukas & Clutton‐Brock, 2012) datasets.

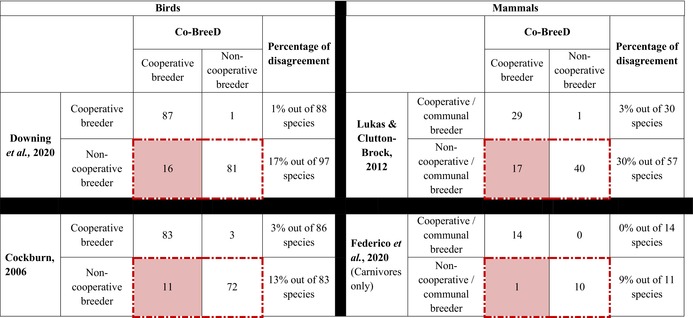

*Note*: Only species included in Co‐BreeD and one of these datasets are included. The cells within the red dashed frames present the number of species classified as non‐cooperative breeders by previous datasets. The red‐filled cells present the number of species for which Co‐BreeD provides new evidence for regular alloparental care.

The discrepancy between Co‐BreeD and previous studies is not due to Co‐BreeD using a broader definition of cooperative breeding and/or alloparental care. First, some of the compared datasets (Federico et al., 2020; Lukas & Clutton‐Brock, 2012) used a narrow definition that only includes species with alloparental care and within‐group reproductive skew. To avoid this bias, Table 2 compares estimates of species with alloparental care (i.e. species classified as cooperative breeders in Co‐BreeD and species classified as cooperative or communal breeders in previous datasets) against estimates of species without alloparental care (i.e. non‐cooperative, nor non‐communal breeders). Second, Co‐BreeD only considered unambiguous forms of alloparental care (e.g. feeding, nursing, transporting of young between locations) that were also used in the compared datasets (Federico et al., 2020; Lukas & Clutton‐Brock, 2012). Rather, the re‐classification of many species as cooperative breeders by Co‐BreeD is mostly the combined result of updated natural history data (e.g. Le Corre et al., 2020; Nyaguthii et al., 2025), reviewing multiple populations per species (e.g. Balmforth, 2004; Millar et al., 1994) and confirmation by species experts.

These results suggest that prior assessments underestimate the prevalence of cooperative breeding in birds and mammals. Specifically, Co‐BreeD presents evidence of cooperative breeding in 35 species that were previously classified as non‐cooperative breeders or that were not included in recent bird (Cockburn, 2006; Downing et al., 2020) and mammal (Federico et al., 2020; Lukas & Clutton‐Brock, 2012) datasets. These include 11 bird species (*Anastomus oscitans*, *Catharacta antarctica*, *Mimus saturninus*, *Stachyris nigriceps*, *Cyanoramphus saisseti*, *Sula sula*, *Melanerpes erythrocephalus*, *Sapayoa aenigma*, *Catharus fuscater*, *Catharus fuscescens*, *Acryllium vulturinum*) and 24 mammal species (e.g. *Callithrix flaviceps*, *Cebuella pygmaea*, *Cebus capucinus*, *Cynictis penicillata*, *Dama dama*, *Homo sapiens*, *Glis glis*, *Physeter macrocephalus*, *Rhinopithecus bieti*, *Rhinopithecus roxellana*, *Saguinus fuscicollis*, *Saguinus weddellii*, *Sapajus nigritus*, *Tadarida brasiliensis*, *Vulpes macrotis*).

### 3.3 Adequate usage of the *PA* dataset

The *PA* parameter is independent of the extent of alloparental care provided to offspring. Hence, populations in which most offspring receive alloparental care may have a high percentage in the *PA* parameter, even if the extent of alloparental care is limited. These cases are especially problematic in group‐living species. For example, ring‐tailed lemurs (Gould, 1992; Sauther et al., 1999) and siamangs (*Symphalangus syndactylus*) (Lappan, 2009) live in groups where offspring receive very little alloparental care. We therefore classified these species as non‐cooperative breeders despite a considerable proportion of breeding events having potential alloparents (Figure 2). Users are thus advised to exercise caution (i) when judging species that are binarily classified as non‐cooperative breeders although >5% of breeding events in their sample included potential alloparents (compare the ‘binary cooperative breeding_SMD’ and ‘percentage breeding events with potential alloparents_PA’ columns) and (ii) when using *PA* estimates where the alloparental care criterion was violated (see ‘violated criteria’ column).

In future, this inconsistency between the prevalence and extent of alloparental care will be mitigated as Co‐BreeD will establish a more comprehensive identification index for cooperatively breeding species by expanding to include datasets dedicated to different forms of alloparental care (Figure 1).

## 4 CONCLUSIONS

1. The Cooperative‐Breeding Database (Co‐BreeD) curates biological estimates that are relevant for cooperative‐breeding research.
2. Co‐BreeD has a sample‐based structure, where each sample is linked to a sampling location and period, enabling (i) linkage with time and space‐specific environmental data from external databases and (ii) exploration of within‐species variability.
3. We hereby publish the initial Co‐BreeD dataset on the Prevalence of breeding events with potential Alloparents (*PA*). This dataset enables the study of cooperative breeding as a continuous trait.
4. Co‐BreeD suggests that cooperative breeding is more prevalent in birds and mammals than previously estimated (Table 2).
5. Finally, Co‐BreeD is an updatable multi‐dataset database, to which new samples and datasets of biological parameters can be added. The corresponding author of this paper aims to update and expand the official Co‐BreeD file and encourages researchers to propose corrections and contribute further data (see Data submission and fair collaboration section or contact the corresponding author).

## AUTHOR CONTRIBUTIONS

*Research design*: Yitzchak Ben Mocha and Michael Griesser. *Funding acquisition*: Yitzchak Ben Mocha, Shai Markman and Michael Griesser. *Project coordination*: Yitzchak Ben Mocha and Maike Woith. *Data collection*: Yitzchak Ben Mocha, Maike Woith, Lucia Bruscagnin, Sophie Scemama de Gialluly and Natalie Kestel. *Contribution of unpublished datasets to Co‐BreeD*: Vittorio Baglione, Laurence Cousseau, Rita Covas, Claire Doutrelant, Roman Gula, Oded Keynan, Ana V. Leitão, Jianqiang Li, Lindelani Makuya, Kyle‐Mark Middleton, Stephen Pruett‐Jones, Carsten Schradin, Jörn Theuerkauf, Miyako H. Warrington, Dean A. Williams and Michael Griesser. *Data analysis*: Yitzchak Ben Mocha and Natalie Kestel. *Data validation*: Yitzchak Ben Mocha, Maike Woith, Shai Markman, Sophie Scemama de Gialluly, Lucia Bruscagnin, Vittorio Baglione, Jordan Boersma, Laurence Cousseau, Rita Covas, Guilherme Henrique Braga de Miranda, Cody J. Dey, Claire Doutrelant, Roman Gula, Robert Heinsohn, Oded Keynan, Sjouke A. Kingma, Ana V. Leitão, Jianqiang Li, Kyle‐Mark Middleton, Stephen Pruett‐Jones, Andrew N. Radford, Carla Restrepo, Dustin R. Rubenstein, Carsten Schradin, Jörn Theuerkauf, Miyako H. Warrington, Dean A. Williams, Iain A. Woxvold and Michael Griesser. *Visualisation*: Yitzchak Ben Mocha, Maike Woith, Lucia Bruscagnin and Natalie Kestel. *Writing of manuscript*: Yitzchak Ben Mocha. *Editing of the manuscript*: Yitzchak Ben Mocha, Maike Woith, Szymon M. Drobniak, Shai Markman, Sophie Scemama de Gialluly, Lucia Bruscagnin, Natalie Kestel, Vittorio Baglione, Jordan Boersma, Laurence Cousseau, Rita Covas, Guilherme Henrique Braga de Miranda, Cody J. Dey, Claire Doutrelant, Roman Gula, Robert Heinsohn, Oded Keynan, Sjouke A. Kingma, Ana V. Leitão, Jianqiang Li, Lindelani Makuya, Kyle‐Mark Middleton, Stephen Pruett‐Jones, Andrew N. Radford, Carla Restrepo, Dustin R. Rubenstein, Carsten Schradin, Jörn Theuerkauf, Miyako H. Warrington, Dean A. Williams, Iain A. Woxvold and Michael Griesser.

## FUNDING INFORMATION

Yitzchak Ben Mocha was supported by the Deutsche Forschungsgemeinschaft (DFG, German Research Foundation) under Germany's Excellence Strategy—EXC 2117‐422037984. Maike Woith, Sophie Scemama de Gialluly and Lucia Bruscagnin were supported by the Young Scholar Fund, parental support and the ZENiT Zukunftskolleg grants awarded to Yitzchak Ben Mocha by the University of Konstanz. Jörn Theuerkauf and Roman Gula received funding from the National Science Centre, Poland (grant no. 2018/29/B/NZ8/023123) and the SAVE Wildlife Conservation Fund. Miya Warrington was supported by an Oxford Brookes Emerging Leaders Research Fellowship. Michael Griesser was supported by a Heisenberg Grant no. GR 4650/2‐1 by the German Research Foundation DFG.

## CONFLICT OF INTEREST STATEMENT

The authors declare no competing interests.

## Supporting information


**Figure S1.** A replica of Figure 2a with all bird names.

**Data S1.** The Co_BreeD_V1 data folder.

## Data Availability

A folder with all the Co‐BreeD project's files (Version 1) is stored in the Zenodo repository (https://doi.org/10.5281/zenodo.14697198).
