# Supplementary material for: An integrative, peer‐reviewed and open‐source cooperative‐breeding database (Co‐BreeD)
Source: J Anim Ecol. 2025 Oct 23;94(12):2597–614. doi: 10.1111/1365-2656.70154 (PMC12673239; doi:10.1111/1365-2656.70154)
Supplement: Supplementary file 1 — Figure S1. A replica of Figure 2a with all bird names. [file JANE-94-2597-s002.pdf]

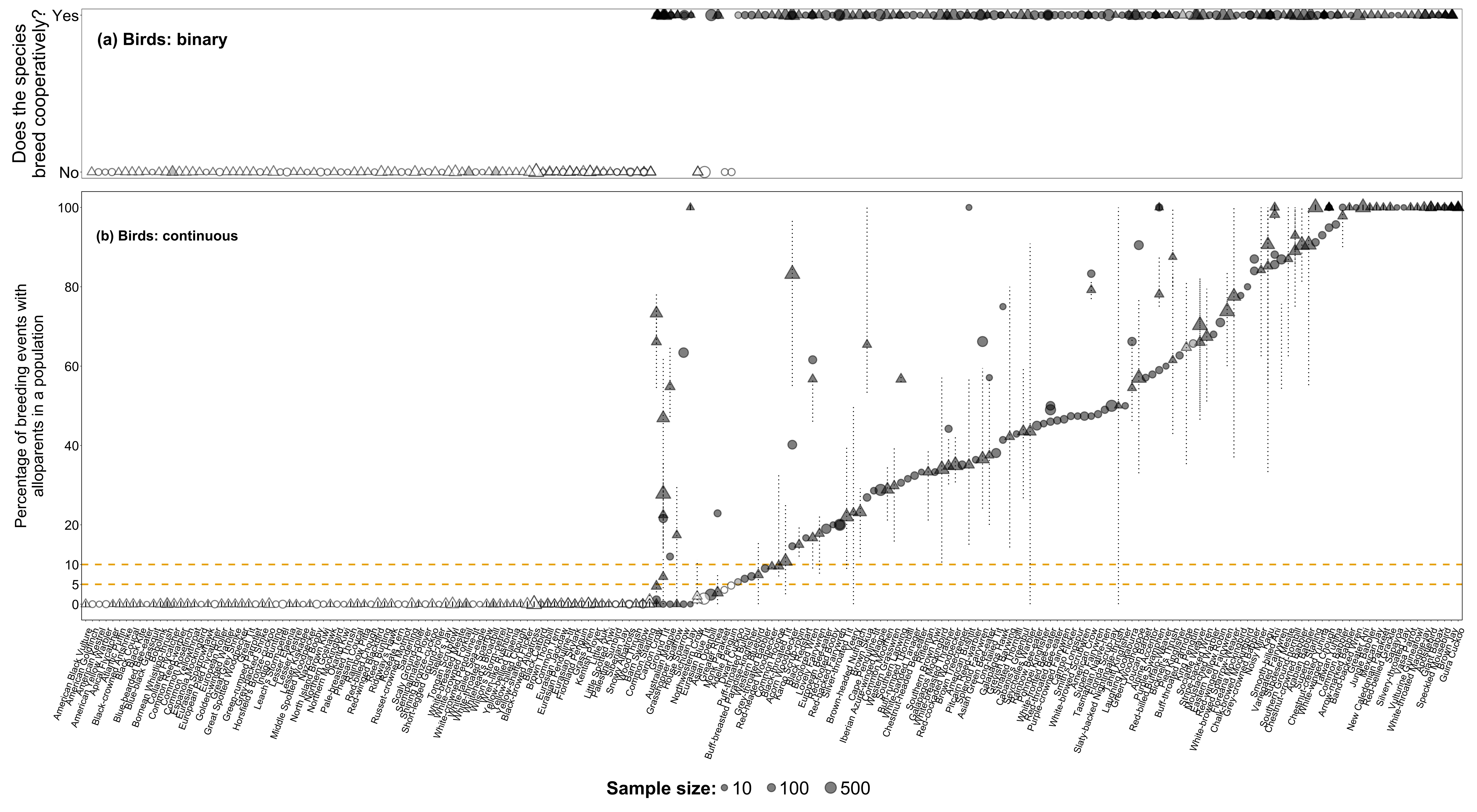
Does the species breed cooperatively?
Yes
No
(a) Birds: binary
Percentage of breeding events with alloparents in a population
100
80
60
40
20
10
5
0
(b) Birds: continuous
Sample size: 10 100 500
